# MZSGO: multimodal zero-shot protein function annotation via evolutionary signals and textual semantics

**DOI:** 10.1093/bioinformatics/btag168

**Published:** 2026-04-03

**Authors:** Boyue Cui, Yujuan Li, Shiqu Chen, Jiaming Wei, Xuan Wang, Yadong Wang, Junyi Li

**Affiliations:** School of Computer Science and Technology, Harbin Institute of Technology (Shenzhen), Shenzhen, Guangdong 518055, China; School of Computer Science and Technology, Harbin Institute of Technology (Shenzhen), Shenzhen, Guangdong 518055, China; School of Computer Science and Technology, Harbin Institute of Technology (Shenzhen), Shenzhen, Guangdong 518055, China; School of Computer Science and Technology, Harbin Institute of Technology (Shenzhen), Shenzhen, Guangdong 518055, China; School of Computer Science and Technology, Harbin Institute of Technology (Shenzhen), Shenzhen, Guangdong 518055, China; Center for Bioinformatics, Faculty of Computing, Harbin Institute of Technology, Harbin, Heilongjiang 150001, China; Key Laboratory of Biological Bigdata, Ministry of Education, Harbin Institute of Technology, Harbin, Heilongjiang 150001, China; School of Computer Science and Technology, Harbin Institute of Technology (Shenzhen), Shenzhen, Guangdong 518055, China; Key Laboratory of Biological Bigdata, Ministry of Education, Harbin Institute of Technology, Harbin, Heilongjiang 150001, China

## Abstract

**Motivation:**

Although deep learning has significantly advanced the field of protein function prediction, current approaches are limited by their reliance on a narrow set of modalities. Specifically, they primarily rely on sequence patterns and treat protein domain data and functional labels merely as categorical tags. Consequently, they fail to capitalize on the semantic richness embedded within their textual definitions. These constraints hinder their ability to generalize to novel labels. To tackle this issue, we present MZSGO, a multimodal zero-shot framework that fuses evolutionary signals from protein language models with semantic features derived from large language models (LLMs). By employing an adaptive gated fusion mechanism, MZSGO effectively aligns sequence-based and text-based modalities to enable robust predictions for unseen labels.

**Results:**

By unifying protein representations and functional annotations, we bridge the semantic gap that limits current approaches. Results indicate that while our model remains competitive on supervised benchmarks, it demonstrates a marked advantage over existing methods in zero-shot tasks. It specifically excels at recognizing previously unseen long-tail and novel Gene Ontology (GO) terms.

**Availability and implementation:**

The source code and datasets are available at https://github.com/toxic-byte/MZSGO.

## 1. Introduction

Proteins play key roles in living systems, directing intricate cellular and molecular processes. Therefore, elucidating protein function constitutes a cornerstone of biology, medicine, and drug discovery (Eisenberg et al. 2000). Despite the exponential growth in protein sequence data driven by high-throughput sequencing technologies, the experimental verification of protein function remains resource-intensive, slow, and expensive. This widening disparity between the abundance of sequences and the scarcity of experimentally validated functions has created an urgent need for robust Automated Function Prediction (AFP) methods (Friedberg 2006). The CAFA challenge (Zhou et al. 2019) has become the premier platform for AFP evaluation, establishing the field’s standardized benchmarking protocols. Such approaches are essential for large-scale protein annotation using GO (Ashburner et al. 2000) classifications across the Biological Process (BP), Molecular Function (MF), and Cellular Component (CC) domains.

Methodologically, AFP approaches have transitioned from single-modality analyses to complex multi-source integration (Lin et al. 2024). Traditional sequence-based methods rely on homology transfer or local motifs, such as Diamond (Buchfink et al. 2021). Network-based methods leverage protein-protein interaction (PPI) networks to infer function based on biological context, while structure-based methods utilize 3D fold information to capture conserved functional signatures. However, as noted in recent comprehensive reviews, relying on a single information source often limits predictive coverage, particularly for proteins with low sequence similarity or incomplete interactome data.

To address these issues, deep learning and hybrid information paradigms have emerged as the dominant approach. Early deep learning models, such as DeepGOCNN (Kulmanov and Hoehndorf 2020), focused on capturing sequence motifs via convolutional neural networks. To further enhance representation power, subsequent researchers began integrating diverse biological features into unified neural frameworks. For instance, Struct2GO (Jiao et al. 2023) incorporates 3D structural features via graph neural networks (Wu et al. 2021), while DPFunc (Wang et al. 2025b) supplements evolutionary sequences with protein domain information to capture modular functional patterns.

Recent research has pivoted toward integrating rich semantic knowledge and multimodal data to improve generalization. DeepGO-SE (Kulmanov et al. 2024) introduced a neuro-symbolic approach combining protein language models with formal axiomatic reasoning to approximate semantic entailment. Addressing the topological complexity of the label space, PO2GO (Li et al. 2024) utilized a novel embedding method, PO2Vec, to capture partial order relationships within the Gene Ontology (GO) directed acyclic graph (DAG) (Goeman and Mansmann 2008). ProtGO (Wang et al. 2025a) established a framework fusing four modalities, including PLM-based representations and explainable text descriptions. Notably, ProtNote (Char et al. 2025) pioneered the application of LLMs for translating amino acid sequences directly into text-based functional descriptions. This approach effectively transforms the complex multi-label classification problem into a set of per-GO-term binary classification tasks.

Despite these significant strides, existing multimodal methods face challenges in the effective fusion of heterogeneous modalities. Methods like ProtGO and DPFunc encode each modality separately, which can limit their ability to model the rich semantic correspondence between sequence features and textual descriptions. Furthermore, while innovative, ProtNote relies on a relatively simple one-hot encoding scheme for sequence representation, which may limit its performance in supervised and zero-shot settings. Moreover, training all ontologies jointly may hamper the model’s capacity to learn features unique to each ontology (see Supplementary Data S1 for detailed experimental validation). In addition, straightforward concatenation of multimodal features can introduce noise, as different modalities contribute information unevenly. To overcome these issues, we introduce MZSGO, a zero-shot protein function prediction framework built on multimodal semantic alignment.

Drawing upon the strategy of ProtNote, we redefine the AFP task as a “Protein–GO” matching problem. MZSGO leverages pre-trained LLMs to extract deep semantic features from GO label definitions and protein domain texts, mapping them alongside protein evolutionary features into a unified latent space. We design an Adaptive Gated Fusion mechanism that dynamically balances the influence of sequence and textual modalities, allowing the model to emphasize the most informative signals. Simultaneously, inspired by dropout regularization (Srivastava et al. 2014), we employ an asymmetric dropout strategy to enhance robustness against modal noise. This design enables MZSGO not only to classify proteins based on learned patterns but also to infer unseen functional labels via semantic similarity.

The main contributions of this work are as follows:

1. MZSGO is a new multimodal AFP framework that improves feature representation by integrating protein sequence information with domain-specific textual descriptions.
2. In MZSGO, LLMs are introduced to construct a semantic space for domains and labels, enabling zero-shot prediction for unseen GO labels and alleviating the limitations of traditional supervised learning.
3. MZSGO proposes an adaptive gated fusion module together with an asymmetric dropout strategy to reduce noise in multimodal inputs. Experiments show that MZSGO achieves superior performance compared with state-of-the-art approaches on standard benchmarks.

## 2. Materials and methods

### 2.1. Overview

**Figure 1:**
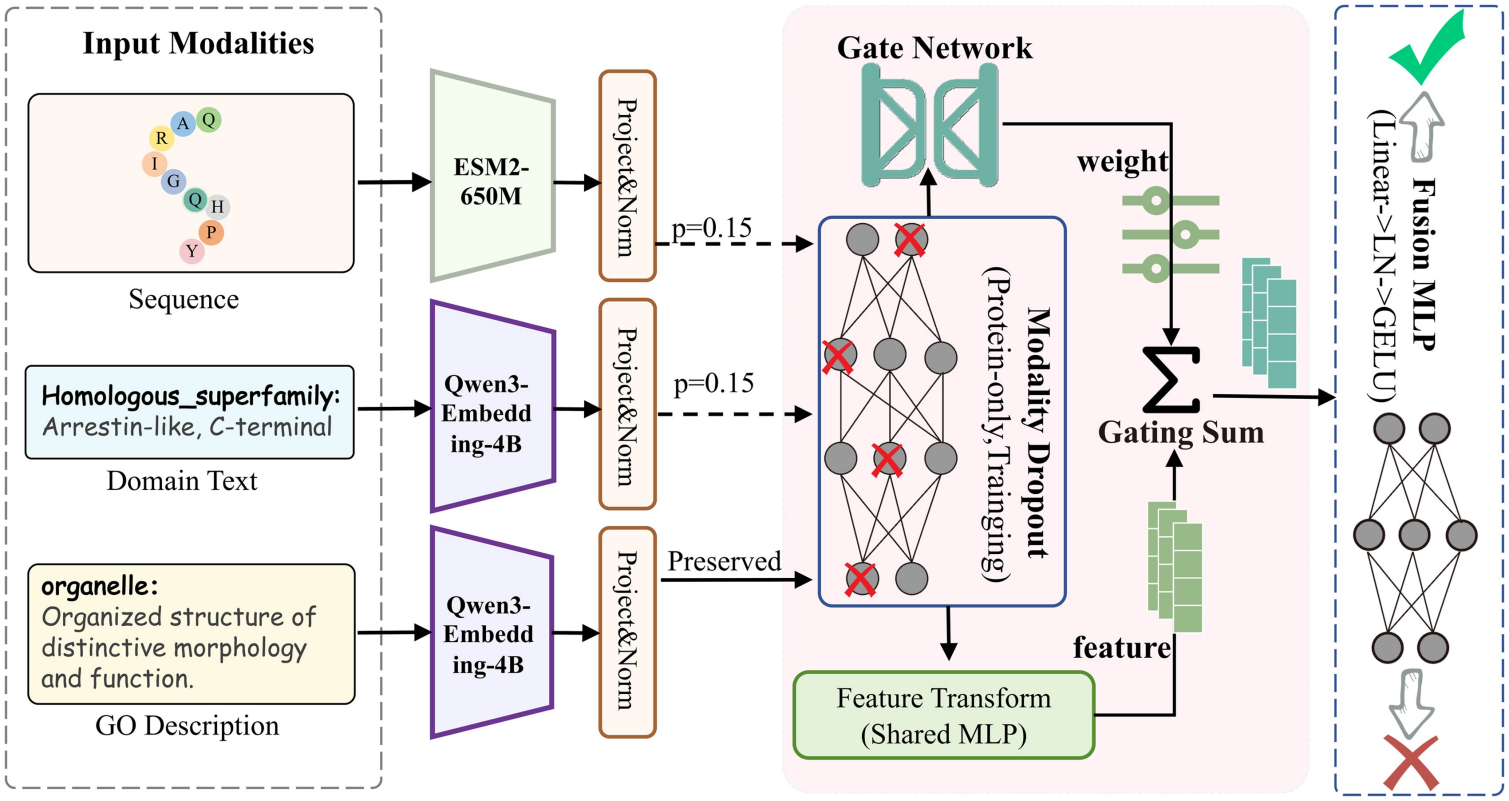
Architecture of the MZSGO framework. The pipeline comprises four integrated stages: (i) multi-modal feature projection; (ii) protein modality dropout during training, where dashed lines (with red crosses) indicate the stochastic discarding of protein features with a probability of P=.15, while solid lines represent preserved modalities; (iii) a gated fusion module that processes the dropped features through two parallel paths: an upper path where features enter a gate network to generate adaptive attention weights, and a lower path where features pass through a shared MLP for transformation. These paths subsequently converge via weighted summation; (iv) a binary classification head for GO function prediction.

The overall structure of our multimodal zero-shot protein function prediction framework is shown in Fig. 1. The system consists of four interconnected components that align biological entities with their functional concepts.

1. *Unified Feature Encoding:* We utilize two pre-trained models to extract features. ESM2-650M (Lin et al. 2023) encodes protein sequences to capture evolutionary patterns. Simultaneously, Qwen3-Embedding-4B (Zhang et al. 2025) serves as a unified semantic encoder, projecting both protein domain textual descriptions (aggregated via mean pooling) and target GO term definitions into a shared latent space.
2. *Asymmetric Feature Dropout:* To mitigate modality overfitting and enhance robustness, we introduce a stochastic dropout mechanism during training. This module randomly masks either the sequence or domain features while preserving the GO embedding, forcing the model to learn effective representations even from partial protein information. This asymmetric design reflects the practical setting of protein function annotation: protein-derived evidence (e.g. domains) may be incomplete or unavailable for some proteins, whereas the textual definition of a candidate GO term is always accessible at inference.
3. *Label-Aware Gated Fusion:* We introduce a gated fusion mechanism that dynamically integrates protein features with functional definitions. Instead of using fixed concatenation, this module assigns adaptive weights to the sequence, domain, and GO embeddings according to their shared context, enabling the model to adjust how much the functional text contributes relative to the biological signals.
4. *Prediction:* The fused multimodal representation is projected through an MLP classifier (Taud and Mas 2017) to generate a compatibility score, predicting the probability that the protein is associated with the specific GO function.

### 2.2. Datasets

To rigorously evaluate the generalization capability of our framework, particularly in zero-shot scenarios, we constructed a comprehensive benchmark based on the CAFA5 challenge and the Swiss-Prot database (Boutet et al. 2007). By incorporating strict homology filtering and temporal partitioning of GO versions, the data construction process simulates the real-world workflow of protein function discovery.

#### 2.2.1. Supervised training set construction

Our training corpus is derived from the CAFA5 dataset, adopting the data source and train/test split strategy utilized in ProtGO, while restricting data entries to those indexed in the Swiss-Prot database. This subset consists of manually reviewed proteins, ensuring high-quality annotations and providing a foundation for fair comparisons with baseline methods that require structural information. We employed the GO version released on 1 January 2023, to retrieve functional annotations. This dataset represents the known functional space, where the model learns to map protein sequences and textual descriptions to existing functional labels.

Distinct from the training protocol of ProtGO, we implemented an ontology-specific filtering strategy. For each ontology category, we exclusively utilized proteins annotated with functions corresponding to that specific category, rather than employing the entire training set directly. This decision was based on the observation that proteins lacking annotations for a specific ontology in the full dataset adversely affect the performance of baseline models. Although our method remains robust on the full dataset without significant performance degradation, we pruned the training set to achieve optimal performance and ensure a rigorous and fair comparison with state-of-the-art baselines. Further details on this construction and the corresponding comparative results are elaborated in Supplementary Data S2.

#### 2.2.2. Zero-shot test set construction

The zero-shot test set is built to assess the model’s capability to annotate proteins it has never encountered and to recognize newly appearing labels. Its design follows two key principles:

1. Protein novelty (sequence level): We selected Swiss-Prot proteins that are absent from the training set. To prevent information leakage caused by sequence similarity, we applied strict homology reduction. Using the Diamond tool, we removed test proteins that had over 30% sequence identity with any sequence in the training set.
2. Label novelty (semantic level): We implemented a temporal evaluation strategy by introducing the ontology updated on October 10, 2025. We define “Zero-shot Labels” as GO terms that exist in the 2025 version but were absent in the 2023 version. Consequently, the final zero-shot test set comprises non-homologous proteins annotated with these newly emerged functions.

#### 2.2.3. Dataset statistics

The dataset statistics are presented in Table 1, with samples grouped into the three GO sub-ontologies: MF, CC, and BP. Due to the complexity of biological pathways, the BP category contains the highest number of zero-shot proteins.

**Table 1:** Dataset statistics.

Dataset	MF	CC	BP
Training set	50 874	51 743	51 618
Test set	5584	5764	5712
Zero-shot	555	17	3687

Table 2 shows the distribution of labels. During the testing phase, we distinguish between three types of labels:

**Table 2:** GO label statistics.

Label category	MF	CC	BP
Total labels	7007	2803	20 639
Unseen labels	191	26	328
Shared labels	2983	1581	11 691
Training-only labels	3833	1196	8620
Zero-shot labels	35	6	34

1. Shared Labels: GO terms that appear in both the training set and the test set.
2. Unseen Labels: Terms existing in the test set but missing from the training data.
3. Zero-shot Labels: Specifically referring to new terms arising from the ontology update.

This label space encompasses all hierarchical levels, ranging from broad functional categories to the most granular leaf nodes present in the annotations. Unseen Labels evaluate the model’s ability to generalize to existing but rare functions excluded from training due to data sparsity, while Zero-shot Labels test the model’s robustness against the dynamic expansion of biological knowledge over time. This rigorous distinction ensures that evaluation metrics on the zero-shot set genuinely reflect the model’s reasoning capability rather than the effects of rote memorization.

### 2.3. Feature extraction stage

The primary goal of the feature extraction stage is to encode diverse biological inputs—protein sequences, domain descriptions, and functional definitions—into dense vector embeddings. Example input data is provided in Supplementary Data S3. We employ advanced pre-trained models to capture the intrinsic features of each modality, avoiding the need to build encoders from the ground up.

#### 2.3.1. Protein sequence representation

To capture the evolutionary signals and physicochemical properties inherent in the primary sequence, we utilize the ESM2-650M model. This Transformer-based (Vaswani et al. 2017) network, pre-trained on millions of sequences, yields high-fidelity embeddings that reflect both protein structure and function. Let a protein sequence be denoted as $P=\{a_{1},\;a_{2},\;\ldots ,\;a_{L}\}$, where L is the sequence length and $a_{i}$ is the specific residue at position i. We feed this sequence into ESM2-650M to extract token-level representations from the last hidden layer. Finally, a global fixed-dimensional representation is obtained by mean pooling all residue embeddings:


(1) $$E_{\text{seq}}=\frac{1}{L}\sum_{i=1}^{L}\text{ESM}(a_{i})$$

where $\text{ESM}(a_{i})\in R^{d_{\mathrm{seq}}}$ is the embedding of the i-th residue, and $E_{\mathrm{seq}}\in R^{d_{\mathrm{seq}}}$ serves as the raw evolutionary feature vector of the protein. We employ mean pooling for its simple implementation, computational efficiency, and ability to ensure fair comparison with existing works that commonly adopt this approach. It is worth noting that more advanced pooling methods have emerged, such as locality-aware pooling (Hoang and Singh 2025), which demonstrate promising prospects in certain application scenarios.

#### 2.3.2. Semantic text representation

To bridge the semantic gap between biological entities and natural language, we employ Qwen3-Embedding-4B, a large-scale text embedding model, as our unified semantic encoder. To obtain fixed-length vectors for functional labels and protein domains, we employ pooling strategies designed to suit the structure of each type of text.

*Label definition encoding:* For each GO term, we utilize its official textual definition as a semantic anchor. Let $T_{\mathrm{GO}}$ be the description text of a specific functional label, consisting of N tokens. The Qwen model outputs a sequence of token embeddings $\left\{h_{1},h_{2},\ldots ,h_{N}\right\}$. We perform a single mean pooling operation over these tokens to obtain the global semantic vector for the label:


(2) $$E_{\mathrm{label}}=\frac{1}{N}\sum_{n=1}^{N}h_{n}$$

where $E_{\mathrm{label}}\in R^{d_{\mathrm{text}}}$ captures the semantic meaning of the function, enabling the model to generalize to unseen labels based on textual similarity. Unlike domains, which must be aggregated to represent a whole protein, each GO term is treated as an independent matching target in our pairwise paradigm, thus requiring only token-level pooling. Derived from official definitions, these semantic anchors provide a static reference space. The model learns to associate protein features with functional semantics—focusing on “what the label means” rather than simply predicting the correct label.

*Domain description encoding:* Proteins often contain multiple functional domains. Let $D=\{d_{1},d_{2},\ldots ,d_{K}\}$ denote the set of domain descriptions associated with a protein, where K is the total number of domains. The domain encoding procedure adopts a two-level mean pooling strategy:

1. Token-level Pooling (First Pooling): For the k-th domain description $d_{k}$ consisting of $M_{k}$ tokens, we first aggregate its token embeddings $\left\{h_{k,1},\ldots ,h_{k,M_{k}}\right\}$ to generate a specific domain embedding $e_{k}$:
  (3) $$e_{k}=\frac{1}{M_{k}}\sum_{m=1}^{M_{k}}h_{k,m}$$
2. Domain-level Pooling (Second Pooling): To form a unified domain feature representation for the entire protein, we aggregate the embeddings of all K domains associated with that protein:
  (4) $$E_{\mathrm{dom}}=\frac{1}{K}\sum_{k=1}^{K}e_{k}$$

where $E_{\mathrm{dom}}\in R^{d_{\mathrm{text}}}$. If a protein has no annotated domains, a zero vector is used as a placeholder. According to the InterPro database (Blum et al. 2025), over 80% of proteins in the UniProtKB database (The UniProt Consortium 2025) have matching domain information, demonstrating the broad applicability of domain-based features in our framework.

### 2.4. Training stage

The objective of the training phase is to learn a mapping function that estimates the likelihood that a protein is linked to a particular GO term. Specifically, during training, each input instance consists of a protein (represented by its sequence and domain descriptions) and a single GO term (represented by its textual definition). Our architecture employs a custom-designed Gated Fusion Network, which adaptively weighs the importance of sequence evolutionary information, structural domain features, and semantic functional descriptions during the decision-making process. MZSGO consists of three domain-specific models (BP, MF, CC), each optimized for its unique biological semantics. Trained via pairwise matching, the model performs inference by scoring protein-GO pairs across the target ontology: for a given protein, we compute its compatibility score with every candidate GO term in the ontology by running the model for each term. This matrix-based approach enables efficient multi-label annotation, the protein and GO term embeddings are computed only once, and the matching scores for all N terms are derived via a single batch operation. The pseudo-code of the training procedure is presented in Supplementary Data S4.

#### 2.4.1. Feature projection and Modality-Specific dropout

Let $E_{\mathrm{seq}}$, $E_{\mathrm{dom}}$, and $E_{\mathrm{label}}$ denote the input embeddings for the protein sequence (ESM), protein domains, and GO labels, respectively. To map these heterogeneous representations into a shared latent space of dimension $d_{\mathrm{model}}$, we employ three independent Multi-Layer Perceptrons (MLPs). Each projection block comprises a linear layer followed by Layer Normalization (LN) (Ba et al. 2016) and a GELU activation (Hendrycks 2016):


(5) $$h_{m}=\text{Dropout}(\text{GELU}(\text{LN}(W_{m}E_{m}+b_{m})))$$

where m∈{seq, dom, label} indicates the modality, and $h_{m}\in R^{d_{\text{model}}}$ is the projected feature vector.

To prevent over-reliance on a single protein modality and simulate missing data scenarios, we introduce a Modality-Specific Dropout strategy during training. We define a binary mask $M=[m_{\mathrm{seq}},\;m_{\mathrm{dom}}]$, where each modality is independently dropped with probability P=.15. Specifically, we sample $r_{\text{seq}},\;r_{\text{dom}}∼\text{Uniform}(0,1)$ and set:


(6) $$m_{\mathrm{seq}}=I[r_{\mathrm{seq}}>p],\;m_{\mathrm{dom}}=I[r_{\mathrm{dom}}>p]$$

The masked features are computed as ${\hat{h}}_{\text{seq}}=h_{\text{seq}}\cdot m_{\text{seq}}$ and ${\hat{h}}_{\text{dom}}=h_{\text{dom}}\cdot m_{\text{dom}}$. To guarantee that at least one protein modality is available, we implement a forced retention mechanism. If both masks are zero ($m_{\mathrm{seq}}=m_{\mathrm{dom}}=0$), we randomly force one to 1:


(7) $$\text{if}\;m_{\mathrm{seq}}=m_{\mathrm{dom}}=0:\;m_{k}\leftarrow 1,\;k∼\text{Uniform}\{\mathrm{seq},\;\mathrm{dom}\}$$

This ensures meaningful input while maintaining regularization. During inference, no dropout is applied, allowing the model to leverage full multimodal information.

#### 2.4.2. Adaptive gated fusion mechanism

To effectively integrate the multimodal features, we design a Gated Fusion Module that learns to assign adaptive importance weights to each information source. First, we concatenate the features to construct a global context vector S, which is passed through a gate network to generate attention scores:


(8) $$S=[{\hat{h}}_{\mathrm{dom}}∥{\hat{h}}_{\mathrm{seq}}∥h_{\mathrm{label}}]$$

(9) $$\alpha =\text{Softmax}(W_{\text{gate}}\cdot \text{ReLU}(\text{LN}(W_{\text{ctx}}S)))$$

where ∥ indicates concatenation. In our implementation, $W_{\mathrm{ctx}}\in R^{d_{\mathrm{ctx}}\times 3d_{\mathrm{model}}\;}a\mathrm{nd}\;W_{\mathrm{gate}}\in R^{3\times d_{\mathrm{ctx}}}$ are learnable weight matrices within the gate network. The resulting 3-dimensional vector $\alpha =[\alpha_{\mathrm{seq}},\alpha_{\mathrm{dom}},\alpha_{\mathrm{label}}{]}^{T}$ denotes the normalized gate weights assigned to the three modalities. Simultaneously, each individual feature is passed through a transformation layer $\phi_{\mathrm{trans}}$, which is implemented as a shared MLP to refine the modality-specific representations. The final fused representation $H_{\mathrm{fused}}$ is obtained via a weighted summation of the transformed features followed by a projection layer $\phi_{\mathrm{proj}}$:


(10) $$H_{\mathrm{fused}}=\phi_{\mathrm{proj}}(\sum_{k=1}^{3}\alpha_{k}\cdot \phi_{\mathrm{trans}}(h_{k}))$$

where k indexes the three modalities (sequence, domain, label), Both $\phi_{\mathrm{trans}}$ and $\phi_{\mathrm{proj}}$ consist of a linear transformation followed by Layer Normalization and an activation function. This mechanism allows the model to dynamically adjust its focus, for instance, by assigning higher weight to domain features when structural motifs are prominent, or relying more on sequence features when domain information is sparse.

#### 2.4.3. Optimization objective

The fused representation $H_{\mathrm{fused}}$ is passed through a final classifier MLP to generate a scalar logit. The probability $\hat{y}$ of the protein possessing the specific function is calculated by the sigmoid function:


(11) $$\hat{y}=\sigma (W_{\mathrm{out}}H_{\mathrm{fused}}+b_{\mathrm{out}})$$

We formulate the protein function prediction problem as a binary classification task. However, to address the varying degrees of class imbalance inherent in different GO sub-ontologies, we employ distinct loss functions for specific branches. For the BP and MF sub-ontologies, we train the model using the standard Binary Cross-Entropy (BCE) loss:


(12) $$L=-\frac{1}{N}\sum_{i=1}^{N}\left[y_{i}l\mathrm{og}({\hat{y}}_{i})+(1-y_{i})\log(1-{\hat{y}}_{i})\right]$$

where $y_{i}\in \{0,1\}$ indicates whether the protein is associated with the corresponding function. For the CC sub-ontology, we adopt the Focal Loss to down-weight the contribution of easy negatives and focus training on hard examples (for a detailed analysis of loss function selection, please refer to Supplementary Data S5):


(13) $$\begin{array}{c} L_{\mathrm{Focal}}=-\frac{1}{N}\sum_{i=1}^{N}[\alpha (1-{\hat{y}}_{i}{)}^{\gamma }y_{i}l\mathrm{og}({\hat{y}}_{i})+(1-\alpha ){\hat{y}}_{i}^{\gamma }(1-y_{i})\log(1-{\hat{y}}_{i})] \end{array}$$

where α is a weighting factor to balance positive and negative classes, and γ is the focusing parameter that modulates the loss for well-classified examples.

## 3. Experimental results and analysis

### 3.1. Metrics for evaluation

To thoroughly assess the effectiveness of our model in protein function prediction, especially its performance in generalized zero-shot settings, we adopt a diverse set of evaluation metrics. In line with the CAFA challenge protocol we primarily report protein-centric Fmax and AUPR to quantify overall performance.

$F_{\max}$ is a threshold-dependent metric. It determines the optimal F1-score by calculating precision and recall values over a range of decision thresholds from 0 to 1, effectively highlighting the model’s peak accuracy. Conversely, the Area Under the Precision–Recall Curve (AUPR) functions independently of specific thresholds. Given the significant class imbalance inherent to functional annotation tasks, AUPR provides a more robust assessment of global performance across all potential thresholds.

To further assess the model’s capability to identify novel functions, we divide the label space into a “Seen Label Set” (present during training) and an “Unseen Label Set” (not observed in training). We calculate seen AUPR and unseen AUPR to evaluate the model’s fit to known functions and its ability to transfer semantic knowledge to new functions, respectively. To assess the balance between known and unknown functions, we adopt the Harmonic Mean (*H*) of seen AUPR and unseen AUPR as a critical metric. A high H value is achieved only when the model performs well on both seen and unseen labels, thereby demonstrating robust generalization capabilities. Comprehensive mathematical definitions for these metrics and experiments evaluating weighted correlation metrics are provided in Supplementary Data S6.

### 3.2. Experimental setup

Our framework was implemented using PyTorch (Paszke et al. 2019). To align heterogeneous modalities, we project the 1280-dimensional ESM embeddings and 2560-dimensional text/domain features into a unified latent space ($d_{\mathrm{model}}=512$). These features are concatenated and processed by the Gated Fusion Network—a two-layer MLP—to compute adaptive modality weights, followed by a projection layer. The fused representation is finally compressed to 256 dimensions for classification.

Training was conducted on a single NVIDIA A6000 GPU (48 GB) using the Adam optimizer (Kingma and Ba 2014) with a learning rate of $5\times 10^{-4}$ and a batch size of 16. To mitigate overfitting, we applied a neuron-level dropout rate of 0.5. Considering the varying complexities of the sub-ontologies, we trained the model for 30 epochs on BP using BCE loss, and 20 epochs on MF (BCE loss) and CC (Focal Loss, γ=2, no α).

### 3.3. Performance comparison

We validate the effectiveness of MZSGO by contrasting its results with those obtained from ten advanced baseline methods. These baseline methods span three major categories: (i) Traditional methods (Naive, Diamond), which rely on label frequency or sequence homology; (ii) Deep learning-based methods (DeepGOCNN, DeepGOZero (Kulmanov and Hoehndorf 2022), Struct2GO, PO2GO, DeepGO-SE), which utilize convolutional networks, graph neural networks, or neuro-symbolic reasoning; and (iii) Multimodal/LLM-based methods (ProtGO, DPFunc, ProtNote), which integrate textual or domain information. Table 3 summarizes the performance comparison for the three ontologies.

**Table 3:** Performance comparison between MZSGO and baseline methods on the test set.

Ontology	Model	**Fmax**↑	**AUPR**↑	**Unseen AUPR**↑	**Seen AUPR**↑	**H**↑
	Naive	0.2991	0.2073	0.0025	0.2076	0.0049
	Diamond	0.4703	0.2913	0.0025	0.2934	0.0050
	DeepGOCNN (2020)	0.3964	0.3676	0.0025	0.3684	0.0050
	DeepGOZero (2022)	0.4888	0.4535	0.0025	0.4558	0.0050
	Struct2GO (2023)	0.4022	0.3744	0.0025	0.3747	0.0050
BP	PO2GO (2024)	0.2975	0.2303	0.0025	0.2307	0.0049
	DeepGO-SE (2024)	0.5006	0.4373	0.0025	0.4404	0.0050
	ProtGO (2025)	0.4636	0.4377	0.0025	0.4410	0.0050
	DPFunc (2025)	0.4984	0.4902	0.0069	0.4936	0.0136
	ProtNote (2025)	0.3555	0.2993	0.0303	0.2997	0.0550
	**MZSGO (Ours)**	**0.5045**	**0.5008**	**0.2393**	**0.5021**	**0.3241**
	Naive	0.5496	0.3318	0.0032	0.3327	0.0063
	Diamond	0.6727	0.3330	0.0032	0.3366	0.0063
	DeepGOCNN (2020)	0.6603	0.5225	0.0032	0.5242	0.0064
	DeepGOZero (2022)	0.7441	0.6333	0.0032	0.6380	0.0064
	Struct2GO (2023)	0.6564	0.5940	0.0032	0.5962	0.0064
MF	PO2GO (2024)	0.5496	0.4136	0.0032	0.4148	0.0064
	DeepGO-SE (2024)	0.7481	0.6332	0.0032	0.6376	0.0064
	ProtGO (2025)	0.7262	0.7399	0.0032	0.7453	0.0064
	DPFunc (2025)	0.7495	**0.7416**	0.0051	**0.7480**	0.0101
	ProtNote (2025)	0.6140	0.5283	0.0490	0.5301	0.0897
	**MZSGO (Ours)**	**0.7611**	0.7337	**0.4806**	0.7380	**0.5821**
	Naive	0.6143	0.0793	0.0207	0.5270	0.0398
	Diamond	0.6331	0.3076	0.0207	0.3093	0.0388
	DeepGOCNN (2020)	0.6918	0.5284	0.0207	0.5292	0.0398
	DeepGOZero (2022)	0.7075	0.6598	0.0207	0.6612	0.0401
	Struct2GO (2023)	0.6989	0.7380	0.0207	0.7382	0.0403
CC	PO2GO (2024)	0.6143	0.5828	0.0207	0.5830	0.0400
	DeepGO-SE (2024)	0.7379	0.6991	0.0207	0.7003	0.0402
	ProtGO (2025)	0.7209	0.7717	0.0207	0.7727	0.0403
	DPFunc (2025)	0.7386	0.7709	0.0274	0.7722	0.0529
	ProtNote (2025)	0.6636	0.6605	0.2530	0.6607	0.3659
	**MZSGO (Ours)**	**0.7470**	**0.7726**	**0.5862**	**0.7733**	**0.6669**

The top-performing results are shown in **bold**, and the runner-up results are underlined.

#### 3.3.1. Performance on general test set

In conventional classification settings (Fmax and AUPR), MZSGO delivers the best results across all three ontologies, highlighting the effectiveness of our multimodal fusion approach.

1. BP Ontology: In the most challenging BP branch, MZSGO achieved an *F*_max_ of 0.5045, surpassing strong competitors such as domain-aware method DPFunc (0.4984) and DeepGO-SE (0.5006).
2. MF and CC Ontologies: Our model maintained its lead with *F*_max_ scores of 0.7611 and 0.7470, respectively.

Unlike single-modality methods, MZSGO effectively integrates protein sequence features (ESM) with domain descriptions via an adaptive gated fusion mechanism. This enables the model to leverage complementary semantic cues, producing stronger feature representations that surpass those of existing methods.

#### 3.3.2. Evaluation of unseen capabilities

A key strength of MZSGO is its ability to infer functions associated with unseen labels. This performance is reflected by the Unseen AUPR and the H reported in Table 3.

Traditional alignment methods and standard deep learning models exhibited limited generalization in unseen scenarios. This is primarily because these models typically treat labels as independent one-hot identifiers. During training, the optimization process suppresses the weights corresponding to unseen labels since they never appear as positive samples, effectively forcing the model to predict a probability of zero for any class not present in the training set. Consequently, the Unseen AUPR values of these approaches are close to the lower bound determined by the evaluation protocol. Notably, although DeepGOZero is theoretically capable of zero-shot prediction via logical constraints, it fails to effectively predict rare classes that lack explicit GO axioms. While ProtNote demonstrates some capability by leveraging LLMs (e.g. 0.0303 in BP), MZSGO outperformed it by a substantial margin. Specifically, MZSGO achieved a Unseen AUPR of 0.2393 in the BP branch—an approximately 8-fold improvement over ProtNote. Consistent trends are observed within the MF and CC ontologies, with MZSGO achieving Unseen AUPR values of 0.4806 and 0.5862, respectively.

The Harmonic Mean (H), which balances performance on seen and unseen classes, further highlights the comprehensive strength of our model. MZSGO achieved H scores of 0.3241 (BP), 0.5821 (MF), and 0.6669 (CC), significantly outperforming the runner-up ProtNote (0.0550, 0.0897, and 0.3659). This confirms that MZSGO does not merely memorize training labels but learns a generalized semantic alignment between protein features and functional descriptions.

#### 3.3.3. Detailed evaluation on zero-shot scenarios

To rigorously validate this generalization ability, we conducted a dedicated experiment on a pure zero-shot test set, comparing MZSGO directly with ProtNote, the strongest baseline for unseen classes. The results are detailed in Table 4. MZSGO consistently outperforms ProtNote in terms of Fmax and AUPR across all three ontologies. A granular analysis of Precision and Recall reveals distinct behavioral differences and improvements:

**Table 4:** Performance comparison on a dedicated true zero-shot test set constructed via temporal evolution.

Ontology	Model	Fmax	AUPR	Precision	Recall
BP	ProtNote	0.3763	0.2705	0.2580	**0.6951**
	**MZSGO**	**0.4881**	**0.4546**	**0.4475**	0.5369
MF	ProtNote	0.3599	0.3393	0.4545	0.2979
	**MZSGO**	**0.5982**	**0.5435**	**0.6261**	**0.5727**
CC	ProtNote	0.2983	0.1652	0.1853	0.7647
	**MZSGO**	**0.6610**	**0.6291**	**0.5521**	**0.8235**

This evaluation assesses the model’s ability to predict GO functional labels that emerged in the 2025 version but were absent from the training cutoff.

1. Balanced Precision-Recall Trade-off in BP: ProtNote exhibits a high Recall (0.6951) but suffers from low Precision (0.2580), suggesting a tendency to overpredict functional labels. In contrast, MZSGO achieves a more balanced trade-off, improving Precision to 0.4475 while maintaining a competitive Recall of 0.5369. This results in a substantially higher Fmax score (0.4881 vs. 0.3763), indicating more reliable predictions for complex biological processes.
2. Comprehensive Dominance in MF and CC: MZSGO demonstrates robust superiority in both the MF and CC ontologies, significantly outperforming baseline methods across all four-evaluation metrics. To further investigate its underlying mechanism, we visualized the functional text embeddings and domain text embeddings for these ontologies. The results reveal that in correctly predicted samples, the domain embedding vectors are spatially closer to the functional text embedding vectors; for a detailed analysis of this phenomenon, please refer to Supplementary Data S7.
3. In MF, MZSGO attained an Fmax score of 0.5982 compared to ProtNote’s 0.3599. Notably, it secures higher Precision (0.6261 vs. 0.4545) and Recall (0.5727 vs. 0.2979), demonstrating its capability to identify unseen molecular functions accurately.
4. In CC, the performance gap is most pronounced. While ProtNote struggles with Precision (0.1853)—likely due to “functional hallucinations” caused by broad textual matching—MZSGO achieves a Precision of 0.5521. It also achieves a superior Recall of 0.8235 (vs. 0.7647). This combination leads to a remarkable Fmax of 0.6610, more than doubling the baseline’s performance (0.2983).

This comparison demonstrates that MZSGO provides highly reliable predictions for novel functional categories. By anchoring predictions in a domain-specific semantic space, MZSGO avoids the noise and false positives common in other text-based models, making it a trustworthy tool for annotating proteins with evolving functional definitions.

### 3.4. Ablation study

To assess the contribution of each component—particularly multimodal features, the fusion module, and the optimization strategy—we perform comprehensive ablations across all three ontologies (BP, MF, and CC). We focus our detailed discussion on the CC ontology here, while the complete ablation results for BP and MF are provided in Supplementary Data S8. Variants fall into five groups: (i) Single-modality encoders (ESM Only, Domain Only); (ii) Feature representation changes (Domain One-hot, PO2Vec label embeddings); (iii) Architectural alterations (Simple Concat, No Feature Drop); (iv) Encoder substitutions [BioGPT (Luo et al. 2022), BiomedBERT (Chakraborty et al. 2020), Qwen3-Embedding-0.6B, ESM2-150M]; and (v) Loss function variants (BCE Loss, Weighted BCE).

As summarized in Table 5, MZSGO achieves the highest overall harmonic mean (*H* = 0.6669), surpassing all ablations in balancing seen and unseen performance.

**Table 5:** Ablation results on the CC ontology.

Method	*F*max	AUPR	Unseen AUPR	Unseen Fmax	*H*
Modality ablation					
ESM Only	0.7319	0.7344	0.5203	0.1793	0.6094
Domain Only	0.7311	0.7052	0.5187	0.3322	0.6012
Feature & architecture ablation					
Domain One-hot	0.7203	0.6731	0.5047	0.3000	0.5889
Use PO2Vec Embedding	0.7406	0.7196	0.3193	0.1818	0.4423
No Feature Drop	**0.7544**	0.6857	0.4898	0.3500	0.5714
Simple Concat	0.7381	0.7009	0.5245	0.3469	0.6000
Encoder variants					
BioGPT	0.7419	0.7198	0.4258	0.2935	0.5351
BioMedBERT	0.7406	0.7218	0.5647	0.3000	0.6337
Qwen3-0.6B	0.7406	0.7282	0.5469	0.2379	0.6247
ESM-150M	0.7385	0.7310	0.3864	0.2333	0.5056
Loss function variants					
BCE Loss	0.7501	0.7143	0.5824	0.2581	0.6421
BCE (Weighted)	0.7199	0.7450	0.5127	0.4444	0.6095
MZSGO (Ours)	0.7470	**0.7726**	**0.5862**	**0.5665**	**0.6669**

The best-performing scores are shown in bold. The reported metrics include overall Fmax and AUPR, as well as zero-shot–specific measures. *H* denotes the Harmonic Mean between seen and unseen performance.

Removing semantic domain information caused the most severe degradation. ESM Only yields a low Unseen Fmax (0.1793), indicating that sequence features alone generalize poorly. Domain Only performs better (0.3322) and Domain One-hot (0.3000) remains far below MZSGO, showing that zero-shot transfer depends on the semantic content of domain descriptions, not mere identifiers.

The fusion mechanism is likewise crucial. Replacing gated fusion with Simple Concat reduces *H* to 0.6000, illustrating that naive concatenation cannot effectively integrate modalities. Using PO2Vec label embeddings also harms zero-shot performance (Unseen AUPR 0.3193). Beyond its dependence on the fixed GO graph topology, PO2Vec embeddings are less aligned with sequence and text modalities, further widening the semantic gap during multimodal fusion.

Regarding optimization objectives, standard BCE Loss achieves a high *F*_max_ (0.7501) but lags in overall AUPR (0.7143). While adding positive weights (BCE Loss Weighted) improves Unseen *F*_max_ (0.4444), it significantly degrades the overall *F*_max_ (0.7199) and *H* score (0.6095). Our adoption of Focal Loss (MZSGO) effectively balances the learning of hard examples, yielding the best trade-off.

Encoder replacements uniformly underperform, confirming that MZSGO’s chosen encoders best capture complementary structure across modalities. Removing feature dropout slightly helps seen-class accuracy (*F*_max_ 0.7544) but lowers zero-shot generalization (*H* = 0.5714), highlighting its regularization benefit.

Overall, zero-shot performance emerges from the combined strengths of semantic domain descriptions, text-based label embeddings, and the adaptive gated fusion module, which together enable robust transfer to unseen GO terms. The extended ablation experiments on the BP and MF ontologies exhibit trends highly consistent with those observed on CC. For a detailed analysis of the adaptive gate weights assigned to different modalities across ontologies, please refer to Supplementary Data S9.

### 3.5. Analysis of parameter sensitivity

**Figure 2:**
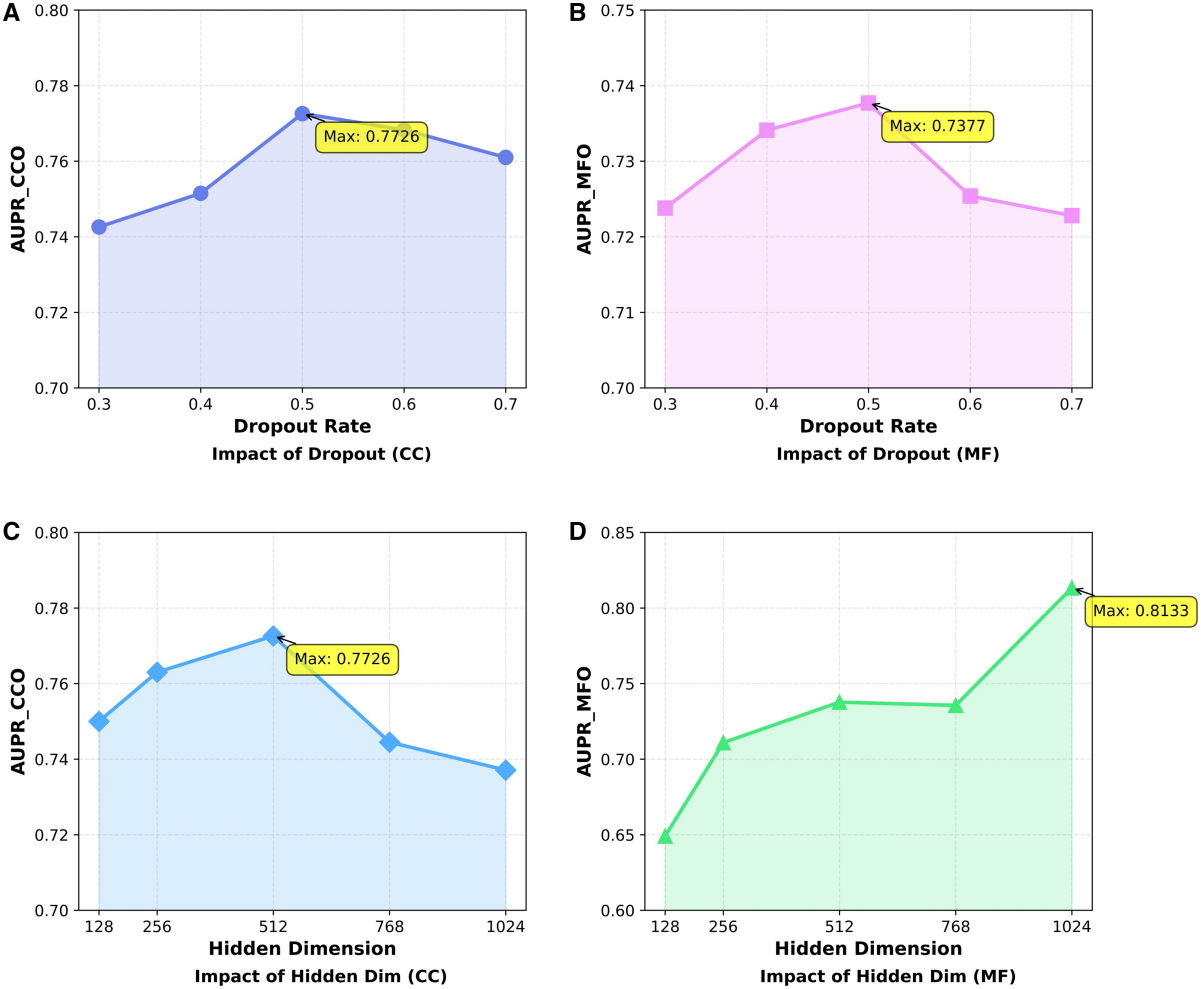
Parameter sensitivity analysis. (A) and (B) Show AUPR with varying dropout rates. (C) and (D) Show the impact of hidden dimension size.

To assess the robustness of our model, we investigated the impact of two critical hyperparameters: the dropout rate and the hidden vector dimension. We adopt a controlled-variable strategy, where one parameter was varied at a time while the others remained fixed. The performance fluctuations, measured by the AUPR across the CC and MF ontologies, are depicted in Fig. 2.

First, we examined the influence of the dropout rate, which is applied after feature extraction to reduce overfitting by randomly disabling a subset of neurons. Dropout values between 0.3 and 0.7 were evaluated. As illustrated in Fig. 2a and b, the model performs best with a dropout rate of 0.5, achieving AUPR scores of 0.7726 on CC and 0.7377 on MF. Lower dropout levels (e.g. 0.3) led to overfitting and weaker generalization, while higher rates (e.g. 0.7) degrade performance (AUPR falls to 0.7610 for CC and 0.7228 for MF) due to excessive information loss. Consequently, a dropout rate of 0.5 is adopted as the optimal configuration.

Subsequently, we evaluated the impact of the hidden vector dimension size, varying it from 128 to 1024. This parameter controls the capacity of the model to represent protein features; a dimension that is too small may fail to capture necessary semantic details, while a dimension that is too large may introduce noise or lead to overfitting. As shown in Fig. 2c and d, expanding the hidden dimension from 128 to 512 leads to a consistent performance gain for both ontologies. For CC, the performance peaks at 512 (0.7726) and subsequently degrades as the dimension increases to 768 and 1024. Interestingly, while the MF ontology shows continued improvement at dimension 1024 (0.8133), the performance on CC drops significantly to 0.7371 at this size. To build a model architecture that generalizes effectively across different GO categories without requiring task‑specific adjustments, we set the hidden vector size to 512, providing a good trade‑off between representational capacity and computational cost.

## 4. Conclusion

In this work, we introduce MZSGO, a multimodal framework for protein function prediction that tackles the challenges of zero-shot generalization through enhanced semantic alignment. This model combines protein evolutionary patterns with high-level semantic representations, integrating the advantages of protein language models and LLMs to effectively predict functions for both seen and unseen labels. Specifically, we utilize ESM to extract evolutionary features and leverage the Qwen3-Embedding model to project protein domain descriptions and GO definitions into a unified semantic space. By employing an Adaptive Gated Fusion mechanism and Asymmetric Dropout, MZSGO dynamically calibrates the contribution of sequence and text modalities, ensuring robust feature learning even in the presence of noise or missing data. The approach presented in this study incorporates rich textual semantics into the AFP task, effectively linking biological entities with natural language descriptions. We evaluated GO consistency by applying the True Path Rule (Notaro et al. 2021) as post-processing (Supplementary Data S10). The resulting negligible performance gains suggest that our multimodal latent space inherently captures hierarchical dependencies, as protein signals and GO definitions naturally align by semantic proximity. By moving beyond traditional classifiers that treat labels as independent IDs, the model can infer functions through semantic similarity. Our comparative analysis demonstrates that MZSGO achieves superior performance. Specifically, in zero-shot scenarios, it significantly outperforms current multimodal and deep learning approaches in terms of both precision and recall.

In future work, we plan to further investigate additional dimensions of protein representation. While MZSGO successfully integrates sequence and textual domain information, the 3D spatial arrangement of proteins remains a critical determinant of function. We plan to incorporate structural features predicted by AlphaFold2 (Jumper et al. 2021)  into our multimodal framework. By fusing 3D structural coordinates with our existing sequence-text alignment, we aim to capture functional motifs that are structurally conserved but divergent in sequence, further enhancing prediction accuracy.

Furthermore, we intend to enhance the model’s capacity for semantic reasoning. While the existing MZSGO approach relies heavily on textual descriptions of GO terms to perform zero-shot inference, this overlooks the structural richness of the GO. Since GO is structured as a DAG containing deep logical dependencies, our future work will focus on fusing these graph-based topological constraints with LLM-derived semantic embeddings. We anticipate that merging explicit logical structures with implicit semantic nuances will yield a label space that is more biologically coherent, ultimately boosting the model’s navigation through intricate functional hierarchies.

## Supplementary Material

btag168_Supplementary_Data

## Code availability

The source code and datasets are available at https://github.com/toxic-byte/MZSGO.
