# Supplementary material for: MZSGO: multimodal zero-shot protein function annotation via evolutionary signals and textual semantics
Source: Bioinformatics. 2026 Apr 3;42(5):btag168. doi: 10.1093/bioinformatics/btag168 (PMC13138841; doi:10.1093/bioinformatics/btag168)
Supplement: btag168_Supplementary_Data [file btag168_supplementary_data.pdf]

Supplementary Material for:

MZSGO: multimodal zero-shot protein function annotation via evolutionary signals and textual semantics

Boyue Cui, Yujuan Li, Shiqu Chen,Jiaming Wei, Xuan Wang, Yadong Wang, Junyi Li

S1 Impact of Joint Training on Model Performance

To validate the hypothesis that training all Gene Ontology (GO) domains jointly compromises the model's capacity to learn features unique to each ontology, we conducted a comparative experiment. We evaluated two architectures, ProtNote and our proposed MZSGO, under two distinct training strategies:

1. **Separate Training:** Models are trained specifically for a single ontology (Biological Process, BP; Molecular Function, MF; and Cellular Component, CC).

2. **Unified Training:** A single model is trained jointly on all three ontologies simultaneously.

Figure S1 illustrates the performance comparison in terms of Fmax scores. As observed, the unified training strategy consistently underperformed the separate training strategy across all ontologies and model architectures.

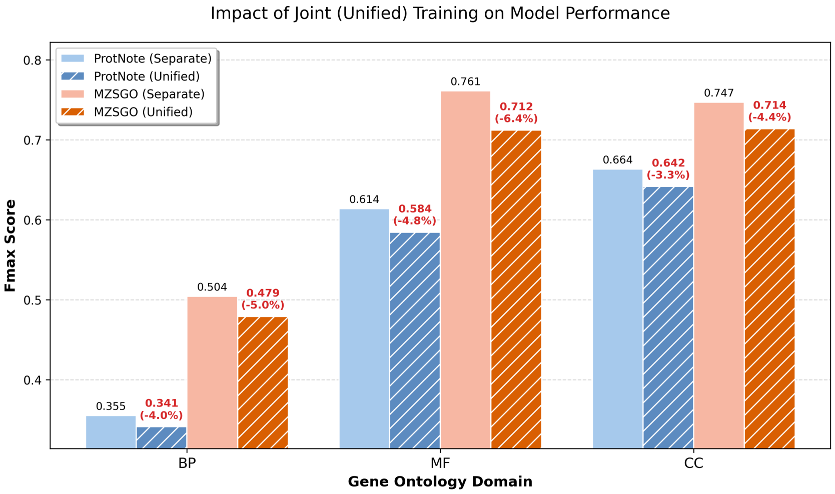

Figure S1: Comparison of Fmax scores between separate and unified training strategies.

S2 Comparative Results without Training Set Filtering

The complete CAFA5 training set within the Swiss-Prot database comprises 72,390 entries. We observed that when the full training set is utilized—which inevitably includes proteins unlabeled for a specific target ontology category—most baseline methods are negatively affected by these implicit "negative" samples, leading to suboptimal performance.

In contrast, methods that cast the protein function task as a binary classification problem (such as ProtNote and our method, MZSGO) demonstrate robustness on the full, unfiltered dataset without significant performance degradation. Figure S2 presents the comparative experimental results without training set filtering. As shown, MZSGO consistently outperforms the latest baselines (ProtGO, DpFunc, and ProtNote) across all three ontology categories in both Fmax and AUPR metrics.

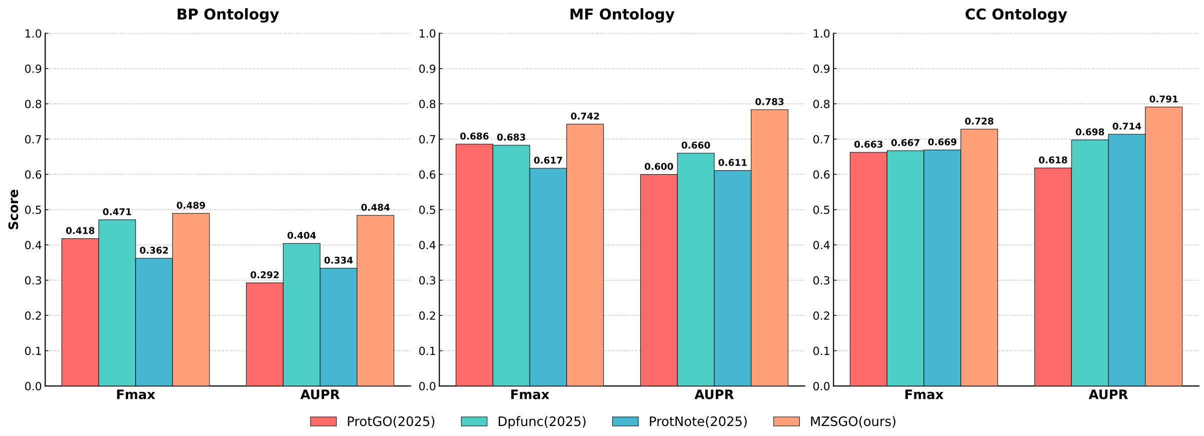

Figure S2: Performance comparison of MZSGO with state-of-the-art methods on the full, unfiltered training set. The evaluation metrics (F-max and AUPR) are reported for BP, MF, and CC ontologies. Our method demonstrates superior performance across all metrics.

S3 Example Input Data

This section presents a complete example of the input data for a single protein entry (UniProt ID: Q8TBH0). Table S1 consists of three modalities: the raw amino acid sequence, domain text descriptions derived from InterPro, and candidate GO term definitions (showing positive samples).

S4 Algorithm Pseudo-code

The detailed training procedure for the MZSGO framework is outlined in Algorithm 1.

Algorithm 1 MZSGO Workflow

Require: E_{esm}, E_{dom} (protein features), E_{nlp} (label features)
Ensure: Prediction Matrix $Y \in \mathbb{R}^{B \times L}$
Define $\phi(\cdot)$: Generic MLP block (Linear $\rightarrow$ LN $\rightarrow$ Act $\rightarrow$ Dropout)
{**1. Projection & Alignment**}
Project features to hidden dimension H:
$\mathbf{h}_{esm} \leftarrow \phi_1(E_{esm}), \quad \mathbf{h}_{dom} \leftarrow \phi_2(E_{dom}), \quad \mathbf{h}_{nlp} \leftarrow \phi_3(E_{nlp})$
Broadcast features to shape $[B \cdot L, H]$ to form protein–label pairs:
$\mathbf{H} \leftarrow \{\mathbf{h}_{dom}^{expand}, \mathbf{h}_{esm}^{expand}, \mathbf{h}_{nlp}^{expand}\}$
{**2. Feature Dropout (Training Only)**}
if Training **then**
  Generate mask $M \in \{0,1\}^{B \cdot L \times 2}$ s.t. at least one protein feature is kept per row.
  $\mathbf{h}_{dom}^{expand} \leftarrow \mathbf{h}_{dom}^{expand} \odot M_{:,0}$
  $\mathbf{h}_{esm}^{expand} \leftarrow \mathbf{h}_{esm}^{expand} \odot M_{:,1}$
end if
{**3. Gated Fusion**}
Concatenate all modalities: $\mathbf{Z}_{cat} \leftarrow \text{Concat}(\mathbf{H}, \text{dim} = -1)$
Compute Gate Weights (Attention):
$\mathbf{W} \leftarrow \text{Softmax}(\phi_{gate}(\mathbf{Z}_{cat}))$ ▷ Shape: $[B \cdot L, 3]$
Weighted Sum Fusion:
$\mathbf{F}_{fused} \leftarrow \sum_{k=1}^{3} \mathbf{W}_{:,k} \cdot \phi_{trans}(\mathbf{H}_k)$
{**4. Classification**}
$Y \leftarrow \text{Reshape}(\text{Linear}(\phi_{cls}(\mathbf{F}_{fused})), [B, L])$
return Y

Table S1: An example input data structure for Protein Q8TBH0.

**Data Modality**	**Content**
**Entry ID**	Q8TBH0
**Modality 1:**<br>Protein Sequence	`MLFDKVKAFSVQLDGATAGVEPVFSGGQAVAGRVLLELSSAARVGALRLRARGRAHVHWTESRSAGSSTAYTQSYSER`<br>`VEVVSHRATLLAPDTGETTTLPPGRHEFLFSFQLPPTLVTSFEGKHGSVRYCIKATLHRPWVPARRARKVFTVIEPVD`<br>`INTPALLAPQAGAREKVARSWYCNRGLVSLSAKIDRKGYTPGEVIPVFAEIDNGSTRPVLPRAAVVQTQTFMARGARK`<br>`QKRAVVASLAGEPVGPGQRALWQGRALRIPPVGPSILHCRVLHVDYALKVCVDIPGTSKLLLELPLVIGTIPLHPFGS`<br>`RSSSVGSHASFLLDWRLGALPERPEAPPEYSEVVADTEEAALGQSPFPLPQDPDMSLEGPFFAYIQEFRYRPPPLYSE`<br>`EDPNPLLGDMRPRCMTC`
**Modality 2:**<br>Domain Text Descriptions	• IPR011021: Domain: Arrestin-like, N-terminal<br>• IPR011022: Domain:Arrestin: C-terminal-like domain<br>• IPR014752: Homologous_superfamily: Arrestin-like, C-terminal<br>• IPR014756: Homologous_superfamily: Immunoglobulin E-set<br>• IPR050357: Family: Arrestin domain-containing protein
**Modality 3:**<br>GO Term Descriptions (positive samples)	**GO:0005622**<br>intracellular anatomical structure: A component of a cell contained within (but not including) the plasma membrane. In eukaryotes it includes the nucleus and cytoplasm.<br><br>**GO:0043226**<br>organelle: Organized structure of distinctive morphology and function. Includes the nucleus, mitochondria, plastids, vacuoles, vesicles, ribosomes and the cytoskeleton. Excludes the plasma membrane.<br><br>**GO:0005575**<br>cellular_component: A location, relative to cellular compartments and structures, occupied by a macromolecular machine when it carries out a molecular function.<br><br>**GO:0016020**<br>membrane: A lipid bilayer along with all the proteins and protein complexes embedded in it and attached to it.

S5 Statistical Rationale for Loss Function Selection

To determine the optimal optimization objectives for BP, MF, and CC, we analyzed their label distributions using the Gini Coefficient (inequality) and Effectiveness Ratio (redundancy).

S5.1 Metric Definitions

Gini Coefficient (G) This metric quantifies distributional inequality. Let x be the label counts sorted in non-decreasing order ($x_1 \leq \cdots \leq x_C$). The coefficient is computed as:

$$G = \frac{2\sum_{i=1}^{C} i \cdot x_i}{C\sum_{i=1}^{C} x_i} - \frac{C+1}{C} \tag{S1}$$

$G \approx 1$ implies maximal inequality, where a few "head" classes dominate the dataset.

Effectiveness Ratio (ρ) Following [1], the marginal benefit of adding samples diminishes due to information overlap. The effective number for a class with n samples is $E_n = (1-\beta^n)/(1-\beta)$, with $\beta = 0.9999$. We define ρ as the ratio of the mean effective count to the mean actual count:

$$\rho = \frac{\frac{1}{C}\sum_{i=1}^{C} E_{n_i}}{\frac{1}{C}\sum_{i=1}^{C} n_i} \tag{S2}$$

A lower ρ indicates high redundancy, where gradients are dominated by repetitive examples.

S5.2 Analysis and Selection

MF and BP exhibit relatively high information quality, with Effectiveness Ratios of 72.20% and 81.11%, respectively. This suggests that despite inherent imbalance, the positive samples provide sufficient unique gradients. Consequently, we employ standard Binary Cross-Entropy (BCE) for these branches.

In contrast, CC demonstrates extreme inequality ($G = 0.946$) and the lowest Effectiveness Ratio (57.60%), significantly worse than BP or MF. This indicates that nearly half of the CC sample volume contributes minimal marginal information. Standard BCE would allow these frequent, redundant examples to overwhelm the learning process. Therefore, we utilize Focal Loss specifically for CC to down-weight easy examples and focus on informative tail classes.

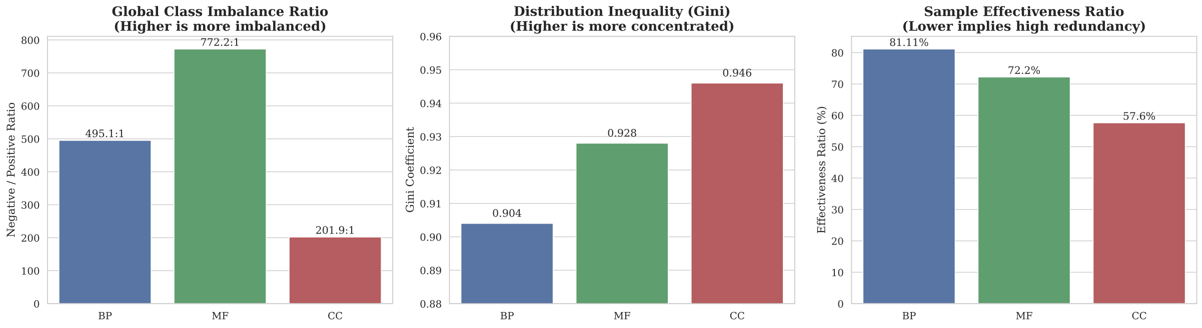

Figure S3: Statistical comparison justifying loss function selection. CC shows the highest inequality and lowest effectiveness, necessitating Focal Loss.

S6 Detailed Evaluation Metrics

In this section, we provide the mathematical definitions of the evaluation metrics used in our study. Following the standard protocols of the CAFA challenge, we employ protein-centric metrics to evaluate the performance of multi-label protein function prediction.

Let N be the total number of protein sequences in the test set. For a given protein i, let T_i be the set of ground truth GO terms (true labels), and let $P_i(t)$ be the set of predicted GO terms with a confidence score greater than or equal to a threshold $t \in [0, 1]$.

S6.1 Standard Protein-centric Metrics

S6.1.1 Precision and Recall

For a specific threshold t, the precision ($\pi_i(t)$) and recall ($\rho_i(t)$) for the i-th protein are defined as:

$$\pi_i(t) = \frac{|P_i(t) \cap T_i|}{|P_i(t)|}, \quad \rho_i(t) = \frac{|P_i(t) \cap T_i|}{|T_i|} \tag{S3}$$

where $|\cdot|$ denotes the size of the set. The average precision ($AvgPr(t)$) and average recall ($AvgRc(t)$) across the dataset are calculated as:

$$AvgPr(t) = \frac{1}{M(t)} \sum_{i=1}^{N} \pi_i(t) \cdot \mathbb{I}(|P_i(t)| > 0) \tag{S4}$$

$$AvgRc(t) = \frac{1}{N} \sum_{i=1}^{N} \rho_i(t) \tag{S5}$$

Here, $M(t)$ represents the number of proteins with at least one prediction at threshold t.

S6.1.2 F-max

The F_{max} score is the maximum harmonic mean of average precision and average recall calculated over all possible thresholds t:

$$F_{max} = \max_{t \in [0,1]} \left(\frac{2 \cdot AvgPr(t) \cdot AvgRc(t)}{AvgPr(t) + AvgRc(t)} \right) \tag{S6}$$

S6.1.3 Area Under the Precision-Recall Curve (AUPR)

AUPR is calculated by integrating the precision-recall curve:

$$AUPR = \int_0^1 AvgPr(Rc)\, d(AvgRc) \tag{S7}$$

Note that in our evaluation protocol, AUPR is computed on protein-centric precision–recall curves over the full label space. Even for models that assign zero scores to unseen labels, the curve still contains non-zero precision–recall points (e.g., at threshold 0). As a result, the numerical AUPR is slightly above zero, although the model has no effective discriminative ability for unseen labels.

S6.2 Generalized Zero-Shot Metrics

To evaluate the model's generalization capability, we split the label space into seen classes (L_{seen}) and unseen classes (L_{unseen}). We calculate the AUPR separately for these subsets:

- **Seen AUPR**: Calculated by restricting the evaluation to the subset of labels present in the training set.
- **Unseen AUPR**: Calculated by restricting the evaluation to the subset of labels never encountered during training.

To assess the trade-off between fitting known knowledge and generalizing to new concepts, we compute the Harmonic Mean (H) of these two metrics:

$$H = \frac{2 \cdot \text{AUPR}_{seen} \cdot \text{AUPR}_{unseen}}{\text{AUPR}_{seen} + \text{AUPR}_{unseen}} \tag{S8}$$

A high H score indicates that the model achieves a balanced performance, avoiding the common pitfall of overfitting to seen classes while failing on unseen ones.

S6.3 Weighted Evaluation Metrics

To account for the varying specificity of GO terms, we employ weighted metrics where each term v is assigned a weight $\tau(v)$ based on its Information Content (IC). The IC is typically defined as $\tau(v) = -\log(p(v))$, where $p(v)$ is the probability of term v occurring in the database.

S6.3.1 Weighted Precision and Recall

For a protein i at threshold t, the weighted precision $w\pi_i(t)$ and weighted recall $w\rho_i(t)$ are:

$$w\pi_i(t) = \frac{\sum_{v \in P_i(t) \cap T_i} \tau(v)}{\sum_{v \in P_i(t)} \tau(v)}, \quad w\rho_i(t) = \frac{\sum_{v \in P_i(t) \cap T_i} \tau(v)}{\sum_{v \in T_i} \tau(v)} \tag{S9}$$

S6.3.2 Weighted F-max (wF_{max}) and Weighted AUPR ($wAUPR$)

Similar to the standard metrics, wF_{max} is the maximum harmonic mean of the weighted precision and recall over all thresholds. $wAUPR$ is the area under the curve formed by weighted precision and weighted recall.

S6.3.3 Semantic Distance (S_{min})

S_{min} evaluates the semantic distance between predictions and ground truth based on information content. It combines Remaining Uncertainty (RU) and Misinformation (MI). For a threshold t:

$$RU(t) = \frac{1}{N} \sum_{i=1}^{N} \sum_{v \in T_i \setminus P_i(t)} \tau(v), \quad MI(t) = \frac{1}{N} \sum_{i=1}^{N} \sum_{v \in P_i(t) \setminus T_i} \tau(v) \tag{S10}$$

The metric S_{min} is the minimum semantic distance observed across all thresholds:

$$S_{min} = \min_{t \in [0,1]} \sqrt{RU(t)^2 + MI(t)^2} \tag{S11}$$

S6.4 Weighted Metric Results

We evaluated our model, MZSGO, against baseline methods (ProtGO, DPFunc, ProtNote) using the weighted metrics. The comparative results for BP, MF, and CC ontologies are summarized in Table S2.

Table S2: Performance comparison using weighted metrics (wF_{max}, $wAUPR$, and S_{min}) across three GO ontologies (BP, MF, CC). All values are reported to 4 decimal places. Best results are highlighted in bold. Note that for S_{min}, lower values indicate better performance.

**Model**	**BP**			**MF**			**CC**		
	**wFmax**	**wAUPR**	**Smin ↓**	**wFmax**	**wAUPR**	**Smin ↓**	**wFmax**	**wAUPR**	**Smin ↓**
ProtGO (2025)	0.4231	0.3582	37.0125	0.6634	0.5312	9.3241	0.6315	0.5553	9.5712
DPFunc (2025)	**0.4562**	**0.4265**	35.4012	0.6912	0.6431	8.6325	0.6521	0.6412	9.0834
ProtNote (2025)	0.3065	0.2384	40.9251	0.5301	0.4105	12.0812	0.5174	0.4776	11.1623
MZSGO (Ours)	0.4560	0.4201	**34.8821**	**0.7015**	**0.6462**	**8.6105**	**0.6573**	**0.6478**	**8.9012**

S7 Visualization Analysis of Domain Embeddings and Protein Function Text Embeddings

As illustrated in Figure S4, the visualization results reveal distinct clustering patterns: in correctly predicted samples, the protein domain embedding vectors are spatially closer to their corresponding functional text embedding vectors. In both the MF and CC ontologies, the Euclidean distances between domain embeddings and functional text embeddings are consistently smaller for correctly predicted cases.

To gain deeper insights into why the model succeeds in these zero-shot predictions, we analyzed the textual correspondence between InterPro domain descriptions and GO term definitions. Figure S5 presents a case study of proteins in the CC ontology. It is observed that in successfully predicted cases, there are many shared or similar keywords between the domain descriptions and the GO term definitions.

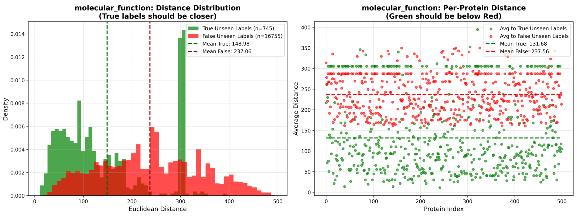

(a) t-SNE Visualization of MF Embeddings

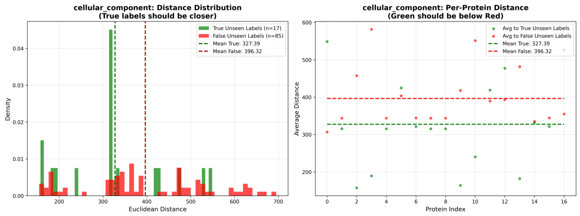

(b) t-SNE Visualization of CC Embeddings

Figure S4: Visualization of Embedding Spaces. (a) and (b) illustrate the t-SNE projections of protein domain embeddings and GO term text embeddings for MF and CC ontologies, respectively. The smaller distance between domains and functions in correctly predicted samples indicates that our method effectively leverages semantic similarity.

Protein ID	True GO Term Description	Domain Annotations	Predicted
Q9VZM7	GO:0160232: INTAC complex: A protein complex containing Integrator and protein phosphatase 2A core enzyme (PP2A-AC) that stably associates with the C-terminus of RNA polymerase II and promotes premature RNA polymerase II transcription termination.	IPR016024 (Homologous_superfamily): Armadillo-type fold<br>IPR026164 (Family): Integrator complex subunit 10	Yes
Q751U7	GO:0180019: Knl1/Spc105 complex: A kinetochore subcomplex that binds to centromeric chromatin and forms part of the outer kinetochore. It helps to recruit outer kinetochore subunits that will bind to microtubules. In humans and yeast respectively, it consists of KNL1/Spc105p and ZWINT/Kre28p.	IPR031361 (Family): Outer kinetochore KNL1 complex subunit Kre28	Yes
Q552L5	GO:0140168: nuclear ribonucleoprotein granule: A ribonucleoprotein granule located in the nucleus.	IPR008501 (Family): THO complex subunit 7/Mft1	Yes
Q54NC8	GO:0160232: INTAC complex: A protein complex containing Integrator and protein phosphatase 2A core enzyme (PP2A-AC) that stably associates with the C-terminus of RNA polymerase II and promotes premature RNA polymerase II transcription termination.	IPR038902 (Family): Integrator complex subunit 1<br>IPR053964 (Domain): Integrator complex subunit 1, R3 domain<br>IPR053965 (Domain): Integrator complex subunit 1, R4 domain<br>IPR053966 (Domain): Integrator complex subunit 1, INTS2-binding domain	Yes
Q6FSC7	GO:0180019: Knl1/Spc105 complex: A kinetochore subcomplex that binds to centromeric chromatin and forms part of the outer kinetochore. It helps to recruit outer kinetochore subunits that will bind to microtubules. In humans and yeast respectively, it consists of KNL1/Spc105p and ZWINT/Kre28p.	IPR031361 (Family): Outer kinetochore KNL1 complex subunit Kre28	Yes
Q6FWW3	GO:0140047: specialized extracellular matrix: Species or cell-type specific extracellular matrices that are different from the two main types of extracellular matrices: the interstitial ECM and the basement membrane ECM in metazoa.	No domains	No
Q75QN2	GO:0160232: INTAC complex: A protein complex containing Integrator and protein phosphatase 2A core enzyme (PP2A-AC) that stably associates with the C-terminus of RNA polymerase II and promotes premature RNA polymerase II transcription termination.	IPR011990 (Homologous_superfamily): Tetratricopeptide-like helical domain superfamily<br>IPR038751 (Family): INTS8<br>IPR057980 (Domain): INTS8, TPR repeats	Yes
Q6CV05	GO:0180019: Knl1/Spc105 complex: A kinetochore subcomplex that binds to centromeric chromatin and forms part of the outer kinetochore. It helps to recruit outer kinetochore subunits that will bind to microtubules. In humans and yeast respectively, it consists of KNL1/Spc105p and ZWINT/Kre28p.	IPR031361 (Family): Outer kinetochore KNL1 complex subunit Kre28	Yes
C5DK07	GO:0180019: Knl1/Spc105 complex: A kinetochore subcomplex that binds to centromeric chromatin and forms part of the outer kinetochore. It helps to recruit outer kinetochore subunits that will bind to microtubules. In humans and yeast respectively, it consists of KNL1/Spc105p and ZWINT/Kre28p.	IPR031361 (Family): Outer kinetochore KNL1 complex subunit Kre28	Yes
Q80V86	GO:0160232: INTAC complex: A protein complex containing Integrator and protein phosphatase 2A core enzyme (PP2A-AC) that stably associates with the C-terminus of RNA polymerase II and promotes premature RNA polymerase II transcription termination.	IPR038751 (Family): INTS8<br>IPR057980 (Domain): INTS8, TPR repeats	Yes
Q54QP0	GO:0140168: nuclear ribonucleoprotein granule: A ribonucleoprotein granule located in the nucleus.	IPR003107 (Repeat): HAT (Half-A-TPR) repeat<br>IPR011990 (Homologous_superfamily): Tetratricopeptide-like helical domain superfamily<br>IPR013633 (Family): siRNA-mediated silencing protein NRDE-2	Yes
Q03508	GO:0170049： box C/D RNP complex： A ribonucleoprotein complex consisting of a box C/D type snRNA and three (Archaea) or four (Eukaryotes) core proteins that have diverse functions, including site-specific methylation of rRNA and processing rRNA.	IPR018812 (Domain): Swiss Army Knife RNA repair protein, HAD domain<br>IPR056904 (Domain): YMR265C-like, C-terminal	No
A0A7J6K629	GO:0160211： apicoplast membrane： Any of the lipid bilayers that surround an apicoplast and form the apicoplast envelope.	IPR011701 (Family): Major facilitator superfamily<br>IPR036259 (Homologous_superfamily): MFS transporter superfamily<br>IPR052983 (Family): Major Facilitator Superfamily Riboflavin Transporter	No
Q55CB6	GO:0160232: INTAC complex: A protein complex containing Integrator and protein phosphatase 2A core enzyme (PP2A-AC) that stably associates with the C-terminus of RNA polymerase II and promotes premature RNA polymerase II transcription termination.	IPR026236 (Family): Integrator complex subunit 2, metazoa<br>IPR029321 (Family): Integrator complex subunit 2	Yes
P82119	GO:0140047: specialized extracellular matrix: Species or cell-type specific extracellular matrices that are different from the two main types of extracellular matrices: the interstitial ECM and the basement membrane ECM in metazoa.	IPR000618 (Family): Insect cuticle protein<br>IPR050468 (Family): Larval/pupal cuticle protein	Yes
Q4V847	GO:0160232: INTAC complex: A protein complex containing Integrator and protein phosphatase 2A core enzyme (PP2A-AC) that stably associates with the C-terminus of RNA polymerase II and promotes premature RNA polymerase II transcription termination.	IPR011990 (Homologous_superfamily): Tetratricopeptide-like helical domain superfamily<br>IPR038751 (Family): INTS8<br>IPR057980 (Domain): INTS8, TPR repeats	Yes
Q54X25	GO:0160232: INTAC complex: A protein complex containing Integrator and protein phosphatase 2A core enzyme (PP2A-AC) that stably associates with the C-terminus of RNA polymerase II and promotes premature RNA polymerase II transcription termination.	IPR029444 (Domain): Integrator complex subunit 5, C-terminal<br>IPR040316 (Family): Integrator complex subunit 5	Yes

Figure S5: Case Study of Zero-Shot Predictions in CC Ontology. This table compares the ground truth GO term descriptions with the InterPro domain annotations of the proteins. The "Predicted" column indicates whether MZSGO correctly identified the label.

S8 Extended Ablation Study on BP and MF Ontologies

To further validate the robustness and generalizability of our framework, we extended the ablation study to the BP and MF ontologies. The experimental settings and variant groupings remain identical to those used for the CC ontology described in Section 3.4 of the main text.

The results, presented in Table S3 (BP) and Table S4 (MF), are highly consistent with our findings on the CC ontology:

- **Necessity of Multimodal Fusion:** Relying on a single modality ("ESM Only" or "Domain Only") leads to a substantial performance drop, particularly in zero-shot metrics (Unseen AUPR and H score).
- **Superiority of Semantic Alignments:** Replacing LLM-based text embeddings with traditional methods ("Domain One-hot" or "PO2Vec Embedding") severely impairs zero-shot performance, reaffirming the importance of deep semantic representations for label understanding.
- **Effectiveness of Architecture:** The "Simple Concat" variant and the "No Feature Drop" setting both underperform compared to MZSGO, proving the necessity of dynamically weighting modalities and utilizing asymmetric feature dropout for regularization.

Table S3: Ablation experiment results on the Biological Process (BP) ontology.

**Model Variants**	**Fmax**	**AUPR**	**Unseen AUPR**	**Unseen Fmax**	**H**
Modality Ablation					
ESM Only	0.4584	0.4278	0.0924	0.0446	0.1520
Domain Only	0.4552	0.4208	0.0774	0.0334	0.1308
Feature & Architecture Ablation					
Domain One-hot	0.4833	0.4550	0.2309	0.1182	0.3066
Use PO2Vec Embedding	0.4948	0.4721	0.0959	0.0534	0.1595
No Feature Drop	0.4992	0.4794	0.2231	0.1440	0.3047
Simple Concat	0.4849	0.4602	0.0700	0.0352	0.1215
Encoder Variants					
BioGPT	0.4877	0.4650	0.1584	0.1153	0.2364
BioMedBERT	0.4829	0.4569	0.1801	0.0931	0.2608
Qwen3-0.6B	0.4842	0.4590	0.1964	0.0967	0.2752
ESM-150M	0.4799	0.4540	0.1730	0.1176	0.2506
Loss Function Variants					
Focal Loss	0.4791	0.4787	0.1166	0.1901	0.1876
BCE (Weighted)	0.4821	0.4580	0.1755	**0.2141**	0.2542
**MZSGO (Ours)**	**0.5045**	**0.5008**	**0.2393**	0.1210	**0.3250**

Table S4: Ablation experiment results on the Molecular Function (MF) ontology.

Model Variants	Fmax	AUPR	Unseen AUPR	Unseen Fmax	H
Modality Ablation					
ESM Only	0.7322	0.6890	0.2502	0.1516	0.3675
Domain Only	0.7380	0.6986	0.4655	0.3966	0.5598
Feature & Architecture Ablation					
Domain One-hot	0.7376	0.7041	0.3149	0.2367	0.4359
Use PO2Vec Embedding	0.7414	0.7014	0.3772	0.3871	0.4916
No Feature Drop	0.7517	0.7104	0.4800	0.4206	0.5740
Simple Concat	0.7446	0.7035	0.3951	0.3050	0.5069
Encoder Variants					
BioGPT	0.7506	0.7223	0.4512	0.4290	0.5566
BioMedBERT	0.7508	0.7206	0.4513	0.4485	0.5562
Qwen3-0.6B	0.7440	0.7111	0.3745	0.3428	0.4915
ESM-150M	0.7497	0.7202	0.4656	0.3527	0.5667
Loss Function Variants					
Focal Loss	0.7280	0.6938	0.4286	0.4835	0.5309
BCE (Weighted)	0.7372	0.7074	**0.5526**	**0.5585**	**0.6234**
**MZSGO (Ours)**	**0.7611**	**0.7337**	0.4806	0.4259	0.5821

S9 Analysis of Adaptive Gate Weights

To understand how MZSGO integrates multimodal information, we analyzed the average gate weights assigned to Domain (α), ESM sequence (β), and NLP text (γ) features across the three GO ontologies. The gate weights are dynamically learned, reflecting the model's reliance on specific modalities for different prediction tasks. Figure S6 illustrates the weight distribution across Overall, Unseen, Seen, and Zero-shot specific label sets.

Consistent with the ablation results in Section 3.4, CC exhibits a heavy reliance on Domain features, with an average weight of 83.60% overall, rising to 91.23% for unseen labels. This suggests that subcellular localization is strongly correlated with specific protein domains and motifs, which provide more generalizable signals than raw sequences for this ontology. In contrast, for BP, the model prioritizes ESM sequence features for seen labels (53.66%), likely because sequence homology is a strong predictor for known functions. However, a significant shift occurs for "Zero" labels (labels with very few or no training samples), where the model increases its reliance on Domain features (from 31.11% overall to 44.76%) and NLP features. This adaptive shift demonstrates MZSGO's ability to leverage semantic domain descriptions when sequence patterns alone are insufficient for rare or novel classes. Finally, MF shows a more balanced distribution between Domain ($\sim$48%) and ESM ($\sim$48%) features, indicating that enzymatic activities and binding functions require both the structural cues provided by domains and the fine-grained residue information captured by the ESM encoder. Overall, the gate weight analysis confirms that MZSGO does not simply average modalities but adaptively selects the most informative representation based on the ontology and the specific nature (seen vs. unseen) of the target labels.

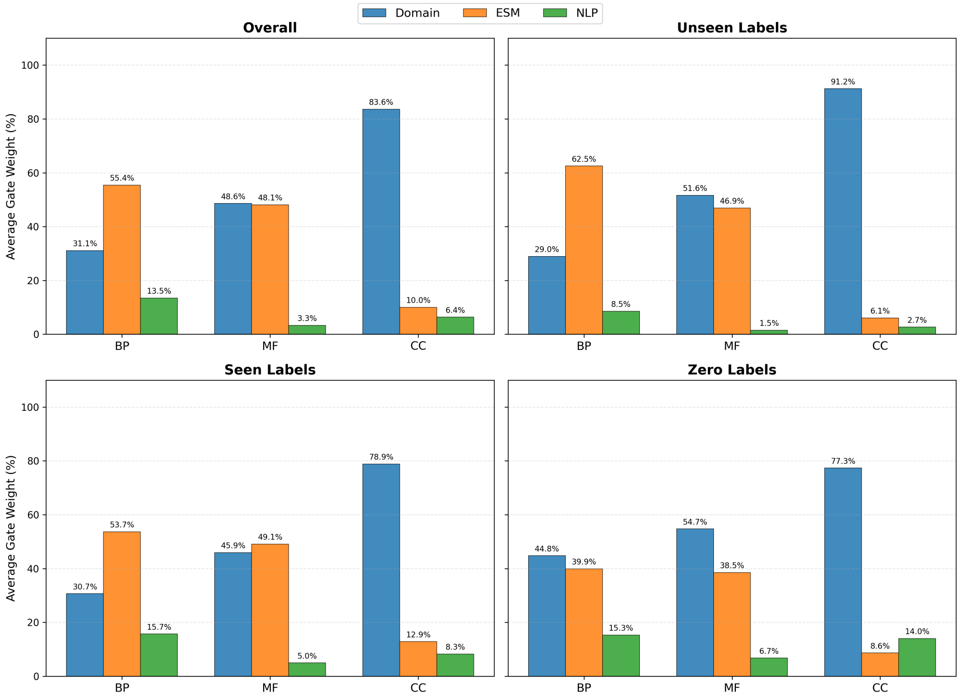

Figure S6: Comparison of adaptive gate weights assigned to Domain, ESM, and NLP modalities across BP, MF, and CC. The subplots show distributions for Overall data, Unseen labels, Seen labels, and Zero-shot labels.

S10 Impact of Hierarchical Consistency and True Path Rule

Gene Ontology (GO) terms are structured as a Directed Acyclic Graph (DAG), which implies the *True Path Rule* (TPR)[2]: if a protein is annotated with a specific term, it must also be annotated with all its ancestral terms. Conversely, if a protein is not annotated with a term, it cannot be annotated with any of its descendant terms.

S10.1 Implementation of Hierarchical Propagation

To evaluate whether explicit hierarchical constraints enhance the performance of MZSGO, we implemented a post-processing propagation mechanism, denoted as **MZSGO_PROP**. Given the raw prediction scores S for a protein across L labels, we ensure hierarchical consistency by propagating the maximum scores from children to parents according to the DAG structure:

$$S_p^* = \max\left(S_p, \max_{c \in \text{children}(p)} S_c\right) \tag{S12}$$

where S_p is the original score for a parent term and S_p^* is the corrected score. This ensures that the prediction score of a parent term is always greater than or equal to the score of any of its descendants.

S10.2 Experimental Results and Discussion

We compared the performance of the original MZSGO (separate training for each ontology) with MZSGO_PROP. The results across Biological Process (BP), Molecular Function (MF), and Cellular Component (CC) are summarized in Table S5.

As observed in Table S5, the integration of explicit TPR propagation leads to marginal improvements (e.g., $< 0.3\%$ in Fmax) across all three ontologies. This finding suggests that MZSGO's multi-modal matching mechanism is inherently capable of capturing hierarchical dependencies. By leveraging LLM-based embeddings of GO definitions, the model learns a latent space where semantically (and thus hierarchically) related terms are positioned closely, naturally leading to consistent score assignments without the need for rigid architectural constraints or complex post-processing.

Table S5: Performance comparison between the original MZSGO and MZSGO with True Path Rule propagation (MZSGO_PROP). The metrics include overall Fmax and AUPR, as well as AUPR for Seen/Unseen labels and the Harmonic Mean (H).

Ontology	Model	Fmax ↑	AUPR ↑	Unseen AUPR ↑	Seen AUPR ↑	H ↑
BP	MZSGO	0.5045	0.5008	0.2393	0.5021	0.3241
	MZSGO_PROP	**0.5066**	**0.5046**	**0.2394**	**0.5060**	**0.3250**
MF	MZSGO	0.7611	0.7337	**0.4806**	0.7380	0.5821
	MZSGO_PROP	**0.7628**	**0.7356**	**0.4806**	**0.7399**	**0.5827**
CC	MZSGO	0.7470	0.7726	**0.5862**	0.7733	0.6669
	MZSGO_PROP	**0.7482**	**0.7736**	**0.5862**	**0.7743**	**0.6672**

References

[1] Yin Cui, Menglin Jia, Tsung-Yi Lin, Yang Song, and Serge Belongie. Class-balanced loss based on effective number of samples. In *Proceedings of the IEEE/CVF conference on computer vision and pattern recognition*, pages 9268–9277, 2019.

[2] Marco Notaro, Marco Frasca, Alessandro Petrini, Jessica Gliozzo, Elena Casiraghi, Peter N Robinson, and Giorgio Valentini. Hemdag: a family of modular and scalable hierarchical ensemble methods to improve gene ontology term prediction. *Bioinformatics*, 37(23):4526–4533, 2021.
